# Lymph Node Lymphatic Endothelial Cell Expansion and Contraction and the Programming of the Immune Response

**DOI:** 10.3389/fimmu.2019.00036

**Published:** 2019-01-25

**Authors:** Erin D. Lucas, Beth A. J. Tamburini

**Affiliations:** ^1^Division of Gastroenterology and Hepatology, Department of Medicine, School of Medicine, University of Colorado Anschutz Medical Campus, Aurora, CO, United States; ^2^Department of Immunology and Microbiology, School of Medicine, University of Colorado Anschutz Medical Campus, Aurora, CO, United States

**Keywords:** lymphatic endothelial cell, lymph node expansion, PD-L1, apoptosis, immune tolerance, lymph node contraction, dendritic cell, interferon

## Abstract

Lymphatic endothelial cells (LECs) form the structure of the lymphatic vessels and the sinuses of the lymph nodes, positioning them to be key players in many different aspects of the immune response. Following an inflammatory stimulus, LECs produce chemokines that recruit immune cells to the lymph nodes. The recruitment of immune cells aids in the coordination of both LEC and lymph node expansion and contraction. More recent data has demonstrated that to coordinate LEC division and death, cell surface molecules, such as PD-L1 and interferon receptors, are required. During homeostasis, LECs use PD-L1 to maintain peripheral tolerance by presenting specific peripheral tissue antigens in order to eliminate tissue specific responses. LECs also have the capacity to acquire, present, and exchange foreign antigens following viral infection or immunization. Here we will review how lymph node LECs require immune cells to expand and contract in response to an immune stimulus, the factors involved and how direct LEC-immune cell interactions are important for programming immunity.

## Introduction

Lymphatic endothelial cells (LECs) are a specialized subset of endothelial cells that comprise lymphatic vessels in the tissue and lymph node (LN). LECs interact with innate and adaptive immune cells both in the tissue and in the LN. LECs have the capacity to produce chemokines in order to recruit immune cells to the LN. Of the chemokines that LECs produce, CCL21 has been implicated in the recruitment of dendritic cells (DC), which in turn promotes LN expansion (1–4). Regulation of LN LEC division and death during LN expansion and contraction is a complicated process to which innate immune cells, adaptive immune cells, and specific signaling molecules contribute. Furthermore, LN LECs not only receive signals from immune cells, but also provide signals to the adaptive immune system to regulate peripheral tolerance and protective immunity. In this review we will highlight how LN LEC interactions and signaling regulate LECs in the LN in response to an inflammatory insult and how LECs program the immune response.

## Regulation of LN LEC Division by the Innate Immune System During Inflammation

During an inflammatory response, the LN must expand to allow for the rapid influx and division of responding lymphocytes. To do this, several coordinated processes in the LN occur: (1) the secretion of chemokines and cytokines and thus the recruitment of innate immune cells; (2) the relaxation of the fibroblastic reticular cell (FRC) network; (3) the division of the stromal cells in the lymph node; (4) the adaptive immune response and (5) the contraction of the LN.

**Figure 1:**
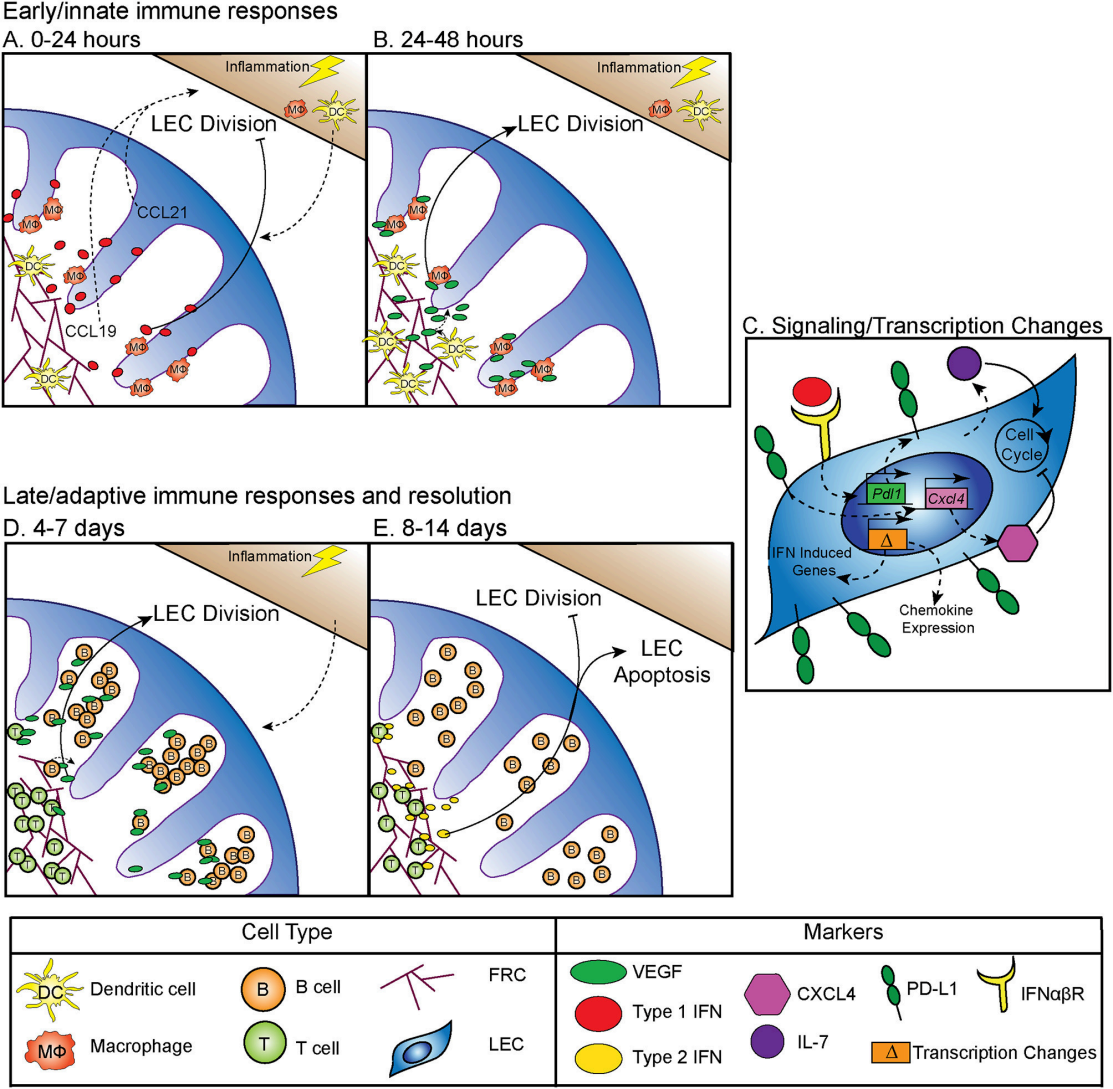
Regulation of LN LEC expansion and contraction during an immune response. **(A)** Control of initiation of LEC expansion by innate immunity: 0–24 h. At early timepoints following an immune stimulus, type 1 IFN production inhibits LEC expansion. DCs traffic to the LN in response to CCL19 and CCL21. **(B)** Control of initiation of LEC expansion by innate immunity: 24–48 h. DCs induce the production of VEGF from FRCs to initiate LEC division. CD11b+ macrophages accumulate along lymphatic vessels in the LN, producing VEGF (5–9). **(C)** Intrinsic mechanisms controlling LEC expansion. LECs produce IL-7, which increases LEC division. PD-L1 expression on LECs inhibits LEC expansion, likely through increasing expression of CXCL4 which is a negative regulator of cell division. PD-L1 expression is controlled by type 1 IFN, resulting in PD-L1 upregulation at early timepoints following an immune stimulus, as well as the expression of other IFN inducible genes. LECs induce expression of chemokines, including CXCL9 and CCL5 following an immune stimulus (5, 10, 11). **(D)** Control of LEC expansion by adaptive immune cells: 4–7 days. At later timepoints during an immune response B cells interact with FRCs, and then produce VEGF to increase LEC expansion. T cells contribute to LEC expansion. **(E)** Control of LEC contraction by adaptive immune cells: 8–14 days. T cells produce type 2 IFN to inhibit LEC expansion and induce LEC apoptosis (8, 12–16).

Between 0 and 24 h following an inflammatory stimulus both type 1 and type 2 interferon (IFN) production is increased, which inhibits LEC division (5) (Figure 1A). Why LEC division is inhibited at this time point is unclear, however this time point coincides with increased expression of CCL19 and 21 by LN stromal cells (17, 18). Dendritic cells (DC) are recruited to the LN through interactions between CCR7 and CCL19 and 21 (19–21) (Figure 1A). Following DC recruitment to the LN, LEC division is initiated. CD11c+ DCs have been shown to lead to LEC proliferation through LEC-DC contact, a process that is ablated following CD11c+ cell depletion (6). Further work showed that DCs regulate the relaxation of the FRC network through the interaction of C-type lectin like 2 (CLEC-2) with podoplanin (PDPN) on the FRCs (22–24). CLEC-2 binding inhibits PDPN signaling, resulting in FRC elongation and increased LN elasticity (24). PDPN expression by LECs and binding by CLEC-2 also elicits the expansion of LECs in the LN (25, 26). In addition to PDPN engagement, DC initiation of LEC proliferation also occurs by inducing vascular endothelial growth factor (VEGF) production by FRCs in the lymph node (8, 9). Early LEC proliferation appears to be independent of T and B cells, as transfer of bone marrow derived dendritic cells into a mouse that lacks T or B cells can elicit LEC expansion at early timepoints following an immune stimulus (6). Thus, DC-stromal interactions are part of the initial step in the expansion of the LN following an inflammatory insult (Figure 1B).

In addition to DCs, macrophages have an important role in regulating lymphangiogenesis in response to inflammation through the production of VEGF, specifically, VEGF-C, VEGF-D, and to a lesser extent, VEGF-A (7). These CD11b+ macrophages accumulate around lymphatic vessels in the draining LN following inflammation in the skin, resulting in LEC proliferation (7). Importantly, following clodronate macrophage depletion, lymphangiogenesis was markedly decreased in the draining LN, demonstrating that macrophages participate in inflammation-induced LEC proliferation (7) (Figure 1B). These data are consistent with macrophages inducing lymphangiogenesis in non-lymphoid organs such as the cornea, peritoneum, and skin (27–29). Neutrophils have been shown to participate in LEC division in the skin (30), both through the production of VEGF-D and by increasing the bioavailability of VEGF-A via MMP-9 and heparanase. However, whether neutrophils or other innate cells contribute to LN LEC expansion is still unclear (30).

Although the mechanisms by which innate immune cells influence LN LEC expansion during an inflammatory response have been fairly well-studied, less is known about the transcription and signaling that occur within the LEC. The primary signals that LECs receive to induce division include VEGF receptor (VEGFR) engagement as described above. However, other factors are involved, including IL-7 which is important for LEC remodeling (11) (Figure 1C). Perhaps not surprisingly, the transcriptional program that ensues in LECs sorted from LNs at 12 h after lipopolysaccharide stimulation suggests that the LECs recruit immune cells through chemokine expression (CXCL9 and CCL5) (10). LECs also increase IFN inducible gene expression (Mpeg1, Lcn2, Irf7, IFI44, and Ly6a among others) at this time point (10). How LN LECs are transcriptionally regulated during inflammation to directly control LEC division could be through the immediate downregulation of genes that regulate cell division, Ccna2 and Klhl9 (10). This downregulation of genes involved in division could be due to the response to IFNγ induced by lipopolysaccharide and may be part of the mechanism behind how IFNα or IFNγ inhibit LEC division (5, 14) (Figures 1A,C). Furthermore, following sorting of LEC populations 6 days after polyI:C injection, non-dividing [(programed death ligand 1(PD-L1^hi^)] LECs express more CXCL4 (an angiostatin), while dividing (PD-L1^lo^) LECs express more growth and differentiation factor 10 (GDF10) and integrin beta 1 (ITGB1), both of which are important for angiogenesis (5, 31–36). Intriguingly, VEGFR3 expression by LECs in the LN was unchanged. However, as LECs generally express high levels of VEGFR3 (10, 37), it seems likely that upregulation of VEGFR3 may not be required to induce LEC division, but instead that LECs require the upregulation of VEGF as described in detail above.

PD-L1 has also been shown to be involved in determining which LECs divide. In both mice that are PD-L1 deficient and mice in which the non-hematopoietic cells lack PD-L1, LEC division was significantly increased at 6 days after polyI:C injection (5). The mechanism behind how PD-L1 could regulate division was at least partially attributed to lost CXCL4 expression in *Pdl1*^−/−^ LECs (5) (Figure 1C). Although CXCL4 was identified as a potential downstream target of PD-L1, much work needs to be done to determine the signaling pathway of PD-L1, and how it regulates division. These new findings indicate that PD-L1 may have a primary function in coordinating LN LEC expansion and survival during inflammation. Therefore, LECs recruit immune cells, receive signals from DCs and macrophages to divide (Figure 1B), change their transcriptional profile and divide based on expression of PD-L1 (Figure 1C) during the early phase (0–48 h) of an inflammatory response.

## Regulation of LEC Expansion by the Adaptive Immune System During Inflammation

While DCs and macrophages contribute to LEC division at early timepoints during an immune response (Figure 1B), B cells have been shown to influence LEC division at the peak of the immune response (Figure 1D). Following immunization with complete Freund's adjuvant, B cell recruitment to the lymph node was required for LEC expansion. In a mouse model where B cells lack L-selectin, an adhesion molecule necessary for lymphocyte migration across high endothelial venules in the LNs, LEC expansion was impaired due to the loss of VEGF-A production in the follicle (12). Intriguingly, utilizing *in vitro* modeling, this group also showed that activated B cells likely produce VEGF-A in the LN only during inflammation (12). Indeed, another study found that inducing the expression of VEGF-A by B cells led to an increase in LN lymphangiogenesis, as well as enlargement of the LN (13). Recently, Dubey et al. showed B cells interact with lymphotoxin-beta receptor (LTβR) on FRCs which results in the production of B cell activating factor (BAFF). In combination with IL-4, production of BAFF causes B cells to produce VEGF-A and C (16). Together, these data suggest B cell production of VEGF-A or C can influence LN LEC expansion, but may not be required (15) (Figure 1D).

Others have shown that in addition to B cells, T cells are also involved in LN and LEC division. First, the lack of both B and T cells led to an almost complete loss of vascular-stromal expansion at later timepoints following complete Freund's adjuvant (8). When only T cells were absent, LEC proliferation was impaired, but surprisingly the absence of T cells did not affect total LEC numbers after complete Freund's adjuvant (8). Other work has also shown a role for T cells in regulating LEC expansion. In a mouse lacking endogenous T or B cells, T cell receptor transgenic T cell transfer did not lead to LEC expansion after immunization, unless the transferred T cells were activated with their cognate antigen (15). Thus, a functional T cell response, in the absence of B cells, is enough to induce LEC expansion following immunization. These data highlight the importance of the adaptive immune response in regulating LEC expansion during late time points (4–7 days) after an inflammatory stimulus (Figure 1D).

## LEC Apoptosis and LN Contraction During Resolution of the Immune Response

While LEC expansion is important for coordinating the immune response, LEC contraction must also occur during the resolution of the immune response. Very little has been done to understand how this process occurs, however, in an athymic mouse, LN lymphatic vessel density is dramatically increased (14). This hypertrophy of lymphatic vessels is reduced by IFNγ production by T cells (14). Furthermore, when IFNγ was absent, lymphatic vessel regression did not occur as it normally does during LN contraction (14). This suggests that the production of IFNγ by T cells may be important for inhibiting lymphatic growth and/or promoting LEC apoptosis (Figure 1E). Interestingly, recent data looking at stromal cells, including LECs, 15 days after lymphocytic choriomeningitis virus, showed increased expression of the chemokines CXCL9 and CXCL10, as well as the activation marker Nur77 (38). While lymphocytic choriomeningitis virus was cleared by this time, LECs remain activated. This could be a process in which LECs recruit IFNγ producing cells until the regression of the lymphatic vasculature and LN size returns to normal.

**Figure 2:**
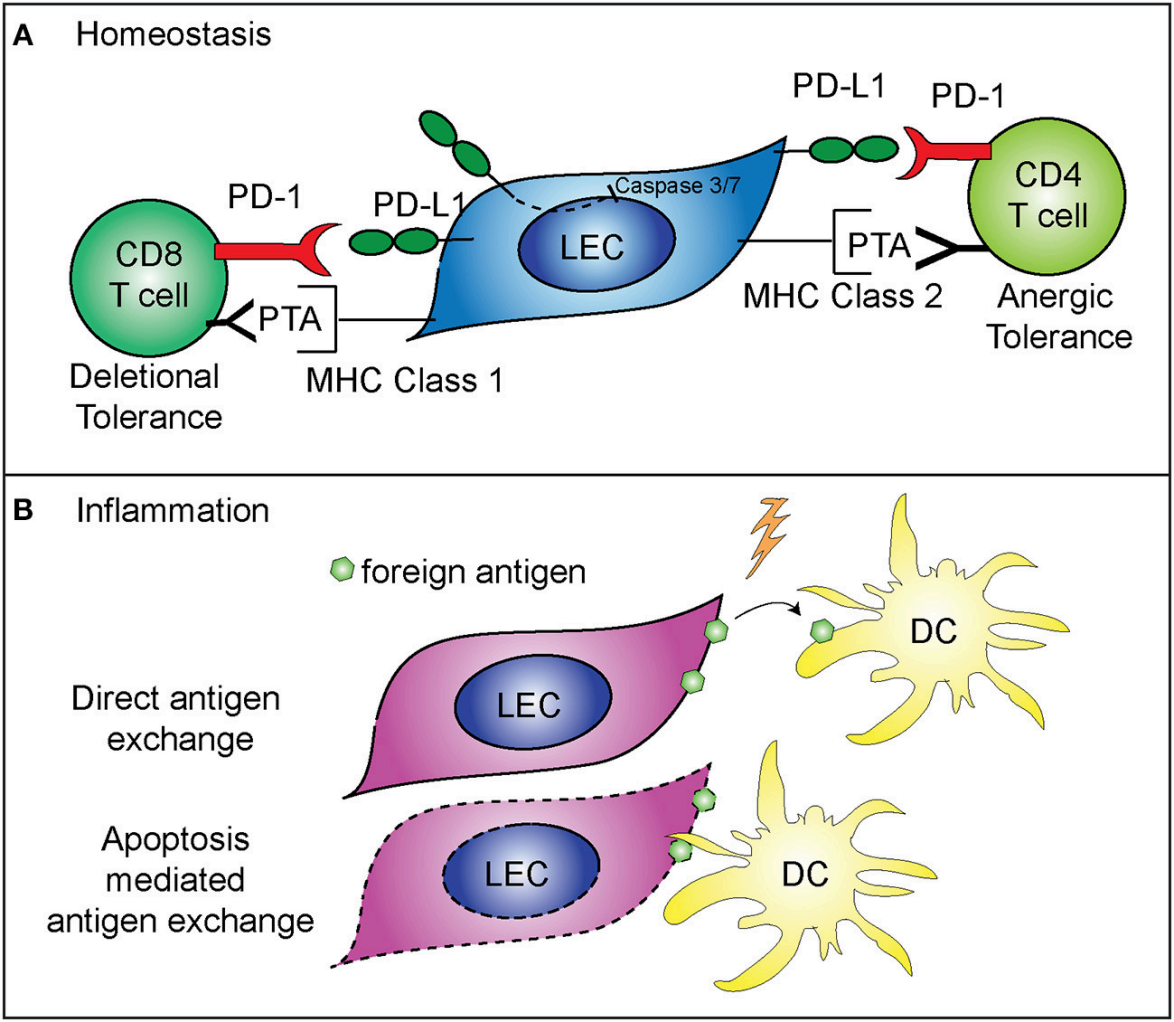
Mechanisms of immune regulation by LECs. **(A)** PD-L1 on LECs inhibits LEC apoptosis and regulates peripheral immune tolerance. PD-L1 negatively regulates cleaved caspase 3/7 production, resulting in decreased apoptosis of LECs that express PD-L1 (5). LECs present peripheral tissue antigens to CD8 T cells on MHC class 1, leading to deletional tolerance via PD-L1 (39, 40). LECs express peripheral tissue antigens (PTA), which are either transferred to DCs for presentation to CD4 T cells or LECs acquire loaded MHC class 2 complexes from DCs, and present to CD4 T cells leading to anergic tolerance via PD-L1 (41, 42). **(B)** LECs archive foreign antigen during inflammation and transfer the antigens to DCs for presentation to memory T cells (15). Two mechanisms are involved in LEC-DC antigen exchange: direct antigen exchange between LECs and DCs, and DC acquisition of archived antigens via LEC apoptosis that occurs during LN contraction (26).

While not directly regulating LEC contraction, PD-L1 does appear to specifically control LEC survival. These findings predict that PD-L1 may determine which LECs undergo apoptosis during LN contraction (5) (Figure 2A). This is consistent with other data showing that PD-L1 can act as a negative regulator of apoptosis in other endothelial cells (43), a process which may be hijacked by cancer cells (44–46). As such, loss of the cytoplasmic domain of PD-L1 in cancer cells resulted in increased apoptosis, from either T cell mediated killing, administration of a chemotherapeutic agent, or interferon beta cytotoxicity (44–46). Although the cytoplasmic domain of PD-L1 is relatively short, it appears that there are at least two signaling domains that help regulate inhibition to apoptosis in response to type 1 interferon, and mutation of these domains can sensitize cancer cells to interferon alpha/beta cytotoxicity (46). While these studies were done in the context of cancer cells which hijack normal cellular functions, recent data suggests that the expression of PD-L1 by LECs, and the regulation of cellular division and survival, may be a normal physiologic role for PD-L1. Further work is needed to determine the precise signaling pathways by which PD-L1 regulates survival, and if this process differs between endothelial cells and cancer cells (Figure 2A).

## LECs Balance Opposing Roles During an Immune Response

While LEC expansion and contraction in the lymph node is important for the immune response, LECs also have a major role in programming the adaptive immune response. As stated above, some LECs in the lymph node express PD-L1 at high levels (47). The role of PD-L1 expression by other cells has been well-described as being inhibitory for T cell activation when programmed death-1 expressed on T cells binds to PD-L1 (48–51). LECs are involved in the maintenance of peripheral T cell tolerance, via expression and presentation of tissue specific antigens, such as tyrosinase (39, 40, 42, 47). Loss of PD-L1 expression by LECs that express tyrosinase results in autoimmune vitiligo (39, 40). In addition, LECs are capable of inducing CD4+ T cell tolerance, through major histocompatibility complex (MHC) class 2. Interestingly, while LECs do not express functional MHC class 2 (42), they are able to acquire loaded MHC class 2—self antigen peptide complexes from DCs (41). These LECs then present the self-antigen to CD4+ T cells which results in anergic self-antigen specific T cells (41). Loss of PD-L1 or loss of LEC acceptance of MHC class 2 complexes leads to autoimmunity (41, 42) (Figure 2A). Furthermore, in tissue lymphatics, PD-L1 also plays a major role in inducing tumor tolerance by LECs (52, 53).

LECs also have a role in the maintenance of protective memory through the archiving of foreign antigens following both immunization and viral infection (15, 26). LECs that acquire antigens during an inflammatory response do not present the foreign antigen directly. Instead, LECs archive antigens, and hand off the antigen to migratory DCs either directly or via LEC apoptosis (Figure 2B). The migratory DCs (26) then present the antigen to CD8+ T cells, improving the effector T cell response upon re-challenge (15). Importantly, antigen archiving is decreased without an inflammatory signal, and other groups have shown that LECs can present foreign antigens in a tolerizing manner when inflammation does not occur, in a process called foreign antigen scavenging (54). In this case, LECs directly present the antigen to CD8+ T cells and the CD8+ T cells are deleted (54). These recent findings illustrate the flexibility of LECs in programming immune responses in the LN and highlight differences between inflammatory and non-inflammatory responses.

What mechanisms regulate the different functions of LECs during the transition from homeostasis to an immune response are not fully understood. An inflammatory signal is needed to prevent LECs from presenting foreign antigen in a tolerizing manner, and a role for LEC expansion has been described in antigen archiving. Following immunization, LECs will acquire, but will not archive antigen unless the LECs are expanding (15). These data indicate that there may be a role for LEC expansion in regulating the function of the lymphatic network during an acute, inflammatory response. What controls LEC expansion during a memory immune response is virtually unknown. However, it seems likely that the immediate production of cytokines, such as IFNγ by memory T cells, would inhibit LEC expansion, similar to the role of effector T cell production of IFNγ inducing LN contraction (14). Interestingly, PD-L1 expression is controlled by both type 1 and type 2 IFN (5), therefore it is possible that, in the absence of division, the upregulation of PD-L1 may be a mechanism to prevent improper activation of auto-reactive T cells and B cells while the LN is preparing to respond to the inflammatory insult (Figure 2).

## Summary

Further research is needed to fully explore novel regulators of LEC expansion and contraction, including PD-L1 and CXCL4. How LECs function to both maintain peripheral tolerance and promote protective immunity is also not well-understood. Understanding these processes, and how LECs can determine the fate of the immune response, are likely very important in the prevention of autoimmunity as well as the development of a strong memory response. Therefore, future studies of LN LECs during an active immune response may lead to novel therapeutic targets in a wide range of diseases.

## Author Contributions

All authors listed have made a substantial, direct and intellectual contribution to the work, and approved it for publication.

### Conflict of Interest Statement

The authors declare that the research was conducted in the absence of any commercial or financial relationships that could be construed as a potential conflict of interest.
